# St. Bartholomew's Hospital

**Published:** 1903-01-24

**Authors:** 


### HOSPITAL ADMINISTRATION.

### CONSTRUCTION AND ECONOMICS.

### ST. BARTHOLOMEW'S HOSPITAL.

In a letter published in the *Times* on the 17th instant, Sir Henry Burdett points out that the population of London is increasing at the rate of 60,000 to 80,000 a year, whilst the plan put forward by Bart.'s to expend £550,000 on a reconstruction scheme when completed would not add a single extra bed to the hospital accommodation of London; that there can be no immediate hurry to come to a decision, seeing that the evidence upon which the present action was taken was largely that given before the Lords Committee in 1890; that in connection with another hospital upwards of 150 years old, where Professor Sims Woodhead recently found micro-organisms present in the wards, ventilators, walls, and floors, Professor Woodhead expressed no surprise, because hospitals built at the date of St. Bartholomew's contain micro-organisms and dust-traps which find no place in a modern hospital. Sir Henry urged the appointment of a small committee to go into the whole question, to study the district of St. Luke's, and, with the assistance of experts, to ascertain (1) the cost of purchasing a site of ample dimensions on which an entirely new hospital may be built, and (2) the time which must elapse before possession can be secured.

#### A Committee Appointed.

At the meeting of the Special Appeal Committee held at the Mansion House on Monday, the 19th instant, Sir Marcus Samuel presiding, there were present:—Sir Trevor Lawrence, Mr. Benjamin L. Cohen, M.P., Mr. J. R. Cooper, Alderman Alliston, Mr. George Baker, Mr. J. C. Lovell, Alderman Sir William Treloar, Mr. Henry Attlee, Sir James Blyth, Mr. P. L. Blyth, Mr. G. A. Davis, Mr. R. W. Edwards, Mr. W. Flux, Mr. James Figgins, Mr. F. M. Fry, Mr. H. J. Gardiner, Dr. Clement Godson, Mr. Bernard F. Harris, Mr. Jackson Hunt, Sir Edward Letchworth, Mr. Henry Perrin, Mr. C. R. Rivington, the Rev. Sir Borradaile Savory, Mr. W. T. Shaw, Sir Thomas Smith, Mr. John Smithers, Mr. J. W. Wheeler-Bennett, Surgeon-General Wicksteed, Mr. Alfred Willett, Mr. Travers B. Wire, and Mr. W. H. Cross, clerk for the Governors of St. Bartholomew's Hospital.

On the motion of Sir Trevor Lawrence, seconded by Mr. Benjamin L. Cohen, M.P., the following resolution was carried unanimously:—

That, having regard to the criticism upon the proposed appeal for the enlargement of St. Bartholomew's Hospital, based on inaccurate information, a committee be appointed to report:—

1. Whether it is desirable in the public interest and on financial grounds to retain St. Bartholomew's Hospital on its present site.

2. In the event of the retention of the hospital on its present site, whether any better scheme of rebuilding than that suggested by the Governors can be devised.

3. Upon any other matters affecting the hospital that the committee may think it desirable to inquire into.

That such committee do consist of 15 members, nine to be nominated by the Lord Mayor and six by the treasurer of the hospital, and that the Lord Mayor and the treasurer be *ex officio* members of the committee.

#### The Present Scheme of Reconstruction Examined.

In the *British Medical Journal* of the 17th inst. is published a block plan of St. Bartholomew's Hospital showing the existing buildings and site, the land recently acquired from Christ's Hospital, and the new buildings which it is proposed to erect. From this plan it is possible to form a fairly accurate idea of the scheme.

Briefly it comes to this: that after expending some £250,000 on additional land and an estimated sum of £300,000 on buildings, the hospital will find itself possessed of a new out-patient department, new pathological department, nurses' home, kitchen offices, quarters for resident medical officers, a house for the vicar, and five new operation theatres; but of not one additional bed for patients. Badly as the hospital is equipped at present, it is, to say the least, astonishing to find that an expenditure of over half a million of money is needed for the purposes indicated.

The plan shows that the four large ward blocks which are arranged round the central courtyard are to be retained, together with the gateway, the church, and the medical school buildings, all other existing buildings being swept away. At the north-east corner of the site are placed the various departments for out-patients, casualties, dispensary, and the steward. Along the southern boundary are four blocks, three of which form the nurses' home (the hospital kitchen being on the top of the centre block), and the fourth is a ward block for the accommodation of patients now treated in a small block at the north-east angle, which is to be pulled down.

At the south-west angle of the site is the pathological building with laboratories, post mortem room, and mortuary and inquest room. Facing Giltspur Street on the west is an isolation block for infectious and septic cases, and on the same street is the vicarage and the quarters for resident medical officers. It is obvious from the plan, not only that the whole site is occupied, not to say crowded with buildings, but that the possibility of extension in the future is practically shut out. The whole length of the boundary between the hospital and the land belonging to Christ's Hospital will almost to a certainty be built upon, and the buildings will without doubt be high ones. Thus sun and air will be to a large extent intercepted just at the point where both would be of the greatest value.

It is of course difficult from a block plan, and without some information as to the relative heights of the buildings, to realise the full effect of the proposed reconstruction; but this much is clear: that the site will be unduly crowded with buildings and that the cost of the alterations is out of all proportion to the accommodation.

One point of detail cannot be passed over. It is proposed as part of the reconstruction scheme to construct a subway connecting the four ward blocks with the three blocks of the nurses' home, the new ward block, and the pathological block. Subways are convenient and sometimes necessary adjuncts, but they are also liable to become serious dangers, and to connect the ward blocks with the pathological department is courting disaster in a way which cannot in any degree be justified.

